# Vasohibins in Health and Disease: From Angiogenesis to Tumorigenesis, Multiorgan Dysfunction, and Brain–Heart Remodeling

**DOI:** 10.3390/cells14110767

**Published:** 2025-05-23

**Authors:** Ghulam Abbas, Annet Kirabo, Usama Ahmed, Jie Liu, Jidong Chen

**Affiliations:** 1 Guangdong Key Laboratory for Biomedical Measurements and Ultrasound Imaging, School of Biomedical Engineering, Shenzhen University Medical School, Shenzhen 518060, China; abbas514@szu.edu.cn (G.A.);; 2 Department of Medicine, Division of Clinical Pharmacology, Vanderbilt University Medical Center, Nashville, TN 37232, USA; 3 Vanderbilt Center for Immunobiology, Vanderbilt University Medical Center, Nashville, TN 37232, USA; 4 Vanderbilt Institute for Infection, Immunology and Inflammation, Vanderbilt University Medical Center, Nashville, TN 37232, USA; 5 Department of Medicine, School of Biomedical Engineering, Shenzhen University Medical School, Shenzhen 518060, China; usama.med.19@gmail.com; 6 College of Life Sciences and Oceanography, Shenzhen University, Shenzhen 518060, China

**Keywords:** vasohibins, angiogenesis regulator, fibrosis TGF-β/SMAD pathway, microtubule detyrosination, cardiac/brain remodeling

## Abstract

Vasohibins (VASHs), comprising VASH-1 and VASH-2, were initially identified as regulators of angiogenesis. Recent studies, however, have unveiled their novel role in fibrosis and microtubule detyrosination. The dysregulated expression of VASHs is associated with several pathological processes, such as angiogenesis dysfunction, microtubule detyrosination, and fibrosis, contributing to various diseases. These findings suggest the pleiotropic effects of VASHs in multiple organs and systems beyond angiogenesis. This review explores the molecular properties of VASHs and their emerging functions in tubulin carboxyl activity and microtubule detyrosination—key to brain and cardiac remodeling. We also discuss the potential therapeutic applications of their interference in diseases such as tumorigenesis, as well as renal-, reproductive-, and liver-related diseases.

## 1. Introduction

Vasohibin (VASH) was first identified and characterized from a newly discovered gene (KIAA1036) in 2004. This discovery marked a significant milestone in angiogenesis research, as VASH was found to be induced by vascular endothelial growth factor (VEGF) in endothelial cells (ECs), which inhibits EC proliferation and migration, resulting in negative feedback against angiogenesis [1]. Since this initial characterization of VASH, it has long been appreciated as a negative feedback regulator in angiogenesis. However, VASH’s function as a detyrosinating enzyme was first reported in 2017 in two different studies, which demonstrated that VASH acts as a tubulin carboxypeptidase (TCP), the enzyme responsible for the detyrosination of α-tubulin [2,3]. This finding expanded the functional range of VASHs beyond angiogenesis, implicating them in microtubule dynamics. Microtubules, assembled from α/β-tubulin polymers, are important parts of the cytoskeleton. They play critical roles in cellular processes, including polarization, chromosomal segregation during cell division, regulating cell motility, maintaining cell shape, and transporting materials [4,5,6,7]. Microtubule post-translational modifications (PTMs) are critical for sub-cellular functioning [8,9], and detyrosination has been a major PTM biomarker for stable microtubules [10,11]. Tubulin tyrosination, catalyzed by tubulin tyrosine ligases (TTL), and detyrosination, mediated by VASHs, represent a reversible cycle that governs microtubule dynamics. Disruptions in this cycle have been linked to severe pathologies, including brain disorders, cancer, and cardiomyopathies [12,13].

VASHs play critical roles in angiogenesis, fibrosis, and microtubule detyrosination. The dysregulation of VASHs results in pathological conditions, such as tumor angiogenesis [14,15], ocular and corneal neovascularization [16], the development of nephropathy [17], diabetic retinopathy [18], age-allied macular disintegration [19], and fibrosis, as well as microtubule-related brain disorganization and cardiomyopathies. Given the importance of the VASH family in disease pathogenesis, as well as the potential to develop therapeutic agents targeting VASHs to treat various ailments (such as cardiac and brain remodeling), we provide a review of the molecular mechanisms and pleiotropic roles of VASHs in health and disease.

## 2. The Molecular Properties of VASHs

**Figure 1:**
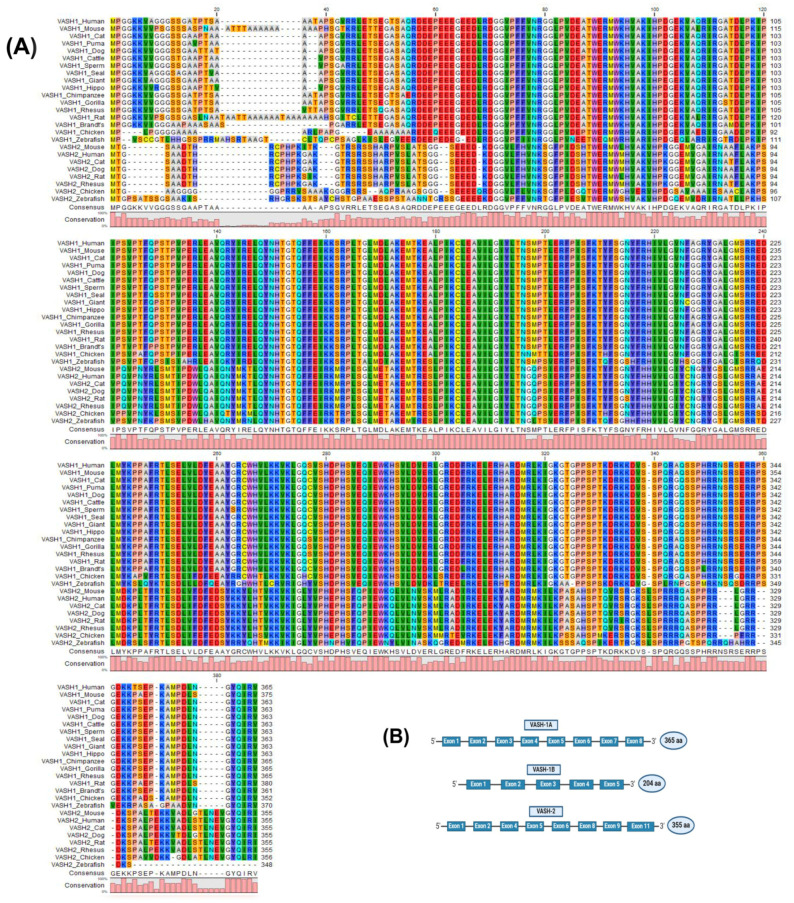
Multiple sequence alignment structure of the vasohibin (VASH) proteins. (**A**) The protein sequences obtained from NCBI (accession numbers provided in Supplementary Materials) were aligned using CLC viewer 8.0. The bar graphs represent the degree of conservation among species, and the colors are in correspondence with the amino acids’ identity. (**B**) VASH-1A contains eight exons and 365 amino acids (aa), whereas VASH-1B contains five exons and 204 aa. VASH-2 has eight exons and consists of 355 aa.

VASH proteins belong to the superfamily of trans-glutaminase-like cysteine proteases and are composed of VASH-1 and VASH-2 [20]. In the human genome, VASH-1 is located on chromosome 14q24.3 and consists of 365 amino acids, whereas VASH-2 is located on chromosome 1q32.3 and consists of 355 amino acids [21,22]. Both paralogs share functional motifs in the primary structure and are highly conserved across different species, as shown in Figure 1A, with approximately 94% of the amino acid sequences of VASHs being identical across species. However, there is a reduced amino acid sequence homology between mice and humans because of variations in the N-terminal portion of VASHs [1]. The primary form of VASH-1 has a molecular weight of 44 kDa, but western blotting analysis revealed multiple bands at 27, 32, 37, and 42 kDa detection, which represent truncated forms. The 42 kDa form has been identified as the secretory and active form [23]. Additionally, there are two VASH-1 splice variants, as follows: VASH-1A, which consists of eight exons, and VASH-1B, which contains the same first five exons as VASH-1A but lacks exons 6–8, which shows reduced potency due to C-terminal truncation [24]. The 11 exons of the VASH-2 gene (Figure 1B) have been shown to generate numerous transcripts for these paralogous genes through alternative splicing [21,24].

The small vasohibin-binding protein (SVBP) has a chaperone-like role in controlling and stabilizing VASH-1 secretion and solubility [25]. VASHs have three conserved domains that physically interact with this chaperone to control the formation of cytosolic punctate structures (PS). As for these functionally critical domains, the VASH-PS domain (residues 91–180 in VASH-1) mediates cytosolic PS formation through its positively charged surface, while the Sla (274–282) and Slb (139–144) motifs are essential for SVBP-dependent dispersion of these structures, and the Slc motif (133–137) stabilizes the VASH-SVBP complex to enable unconventional secretion [26]. Recently, a higher-resolution crystal structure of VASH-SVBP complex isotypes was obtained to define the global organization of the protein [27]. In general, the main VASH domain is divided into three subdomains known as (1) an N-terminal domain (residues 1–120 in VASH-1) that binds SVBP; (2) a central catalytic domain (residues 121–280) harboring the conserved Cys-His-Ser triad, essential for detyrosination activity; and (3) a C-terminal domain (residues 281–365) involved in substrate recognition and microtubule binding. SVBP stabilizes this architecture by sandwiching between the N-terminal and catalytic domains, as determined by X-ray crystallography [28,29,30].

## 3. VASHs and Angiogenesis

**Figure 2:**
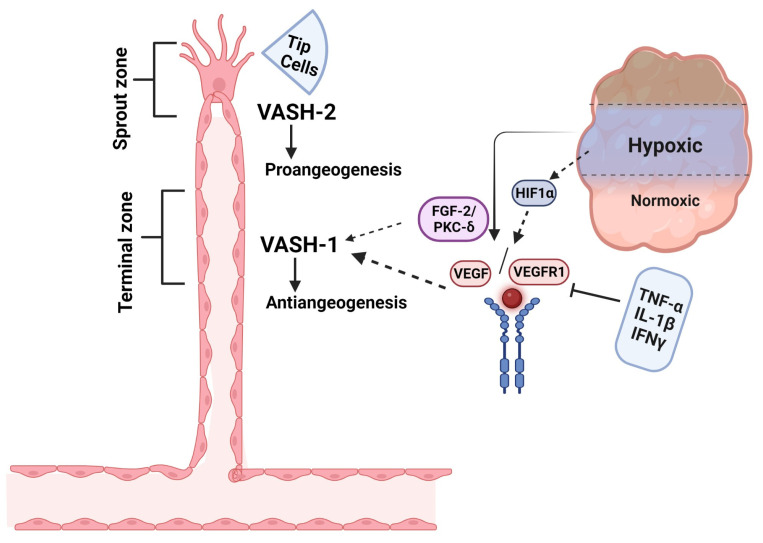
Schematic representation of basic induction of VASH isoforms in angiogenesis regulation. VASH-1 is induced in the vascular termination zone, where it functions to stop angiogenesis, whereas VASH-2 localizes to the sprout zone to promote vessel growth. Under hypoxic conditions, HIF-1α-mediated upregulation of VEGF drives angiogenic activation, which is counterbalanced by VASH-1 through two key mechanisms: (1) FGF2-induced PKC-δ activation and (2) synergistic action with anti-angiogenic cytokines (TNF-α, IL-1β, IFN-γ) to suppress VEGF signaling. This spatial and functional segregation of VASH isoforms creates a dynamic regulatory system for controlled vascular patterning. This figure is adopted from Du et al. [21], licensed under CC BY-NC 3.0. Created in biorender.com.

Angiogenesis is a process in which new blood vessels grow from the existing vasculature [31,32]. The major steps involved in angiogenesis are EC proliferation, migration, vessel remodeling, tube formation, and sprouting. Angiogenesis is regulated by complex mechanisms, including a dynamic balance of both pro- and anti-angiogenic factors. Growth factors such as VEGF, fibroblast growth factor-2 (FGF-2), transforming growth factor (TGF), and cytokines are the main stimulators of angiogenesis, whereas hormones, chemokines, and proteins deposited in the extracellular matrix (ECM) are the main angiogenesis inhibitors [33,34]. Most angiogenesis inhibitors are not produced by the vasculature, rather, they are produced in response to certain stimuli, with some constitutively expressed inhibitors acting as barriers to stop sprout invasion [35,36]. The VASH-1 protein is widely expressed in the ECs of growing mouse, human, and chicken embryos. However, its expression was downregulated in the postnatal period, suggesting a critical role in vascular development [22,37]. VASH-1 expression is regulated by a variety of factors, such as the angiogenic agents VEGF/VEGFR2 and FGF-2, which induce VASH-1 mRNA expression via the activation of the protein kinase C-delta (PKC-δ) pathway [38]. Certain inflammatory cytokines, such as tumor necrotic factor-alpha (TNF-α), interleukin-1β (IL-1β), and interferon-γ (IFN-γ), can decrease the VEGF-induced VASH-1 expression in ECs [39]. Functionally, VASH-1 inhibits EC proliferation, migration, and capillary tube formation [40]. This phenomenon may be mediated by repressing the expression and phosphorylation of VEGFR2, which plays an essential role in angiogenesis [15]. For example, VEGF upregulates VASH-1 in the retina, which in turn suppresses VEGFR2 and retinal neovascularization [41]. Paradoxically, VASHs can have opposing roles in regulating angiogenesis. For example, VASH-1, which is expressed in the vascular termination zone, inhibits angiogenesis; however, VASH-2, which is primarily found in the vascular sprout zone, exerts an opposing effect by stimulating angiogenesis (Figure 2) [21]. The predominant expression of VASH-2 is reported within the mononuclear cells, mobilized from the bone marrow sprouting front, thus facilitating the angiogenesis [42]. This VASH-2 expression appears constitutively and is not induced by cytokines or growth factors [22,43]. VASHs have a critical role in angiogenesis, serving as critical modulators in several physiological and pathological processes related to angiogenesis.

### 3.1. Angiogenic Role of VASHs in Tumorigenesis

**Figure 3:**
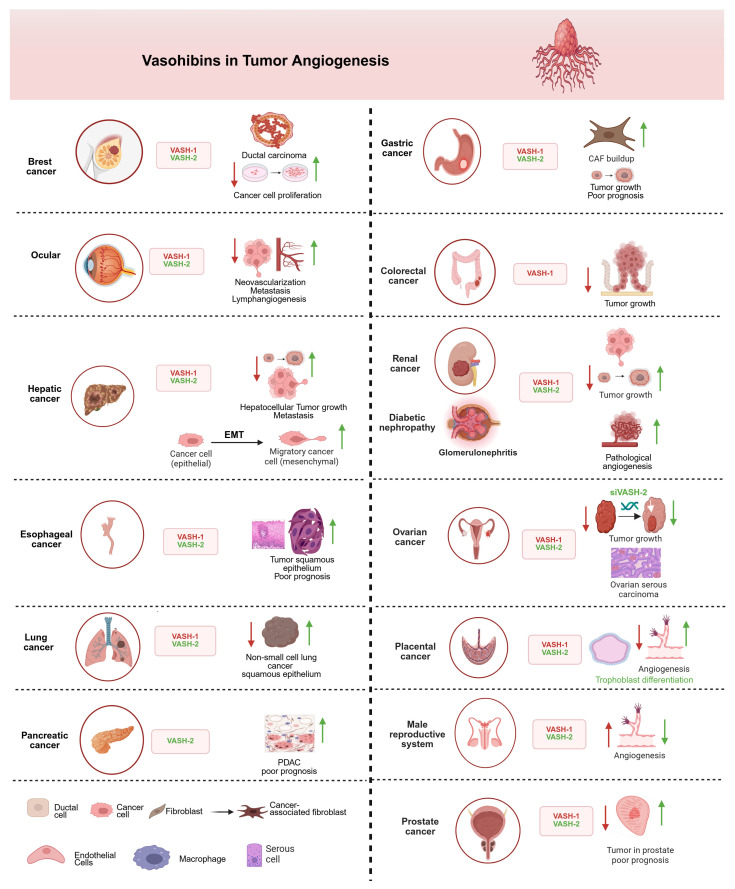
The involvement of VASHs in different types of tumors and organ systems. VASH-1 acts as an anti-angiogenetic, inhibiting/downregulating factor (red arrow), and VASH-2 acts as pro-angiogenic, activating/upregulating factor (green arrow). VASH-1 consistently suppresses tumor progression through angiogenesis inhibition in most cancers, while VASH-2 promotes vascular growth and tumor development, except for the male reproductive system, where VASH-1 paradoxically enhances angiogenic processes to rescue erectile dysfunction. In renal cancer, VASH-2 promotes glomerular damage via aberrant angiogenesis and primary tumor growth, whereas VASH-1 downregulation enables renal cancer metastasis—a duality suggesting isoform-specific therapeutic targeting. The epithelial–mesenchymal transition (EMT) panel highlights VASH-2’s role in promoting cancer cell plasticity. All depicted interactions are supported by experimental evidence discussed in the main text. Created in biorender.com.

Clinical evidence demonstrates VASH-1’s therapeutic potential across multiple tumors, suggesting that VASH-1 may inhibit carcinogenesis. Indeed, VASH-1 prevents tumor growth and metastasis by inhibiting tumor angiogenesis in animal tumor models [44,45]. For example, in breast ductal carcinoma, VASH-1 overexpression reduced tumor microvessel density by 58% and decreased xenograft growth by 42% [46]. In addition, ocular studies showed recombinant VASH-1 could reduce pathological choroidal neovascularization by 73% [16]. Similarly, in non-small-cell lung cancer, high VASH-1 expression correlated with significantly better patient survival (hazard ratio = 0.41) [47]. In addition, VASH-1 also plays role in the esophagus [48], liver, pancreas [49], stomach [50,51], colon [52], kidney [53], ovary [54], placenta [55], prostate [56], and male reproductive organs [57] (Figure 3).

In contrast to VASH-1, VASH-2 has been shown to promote tumor growth [58]. For instance, increased VASH-2 expression in cancer fibroblasts promotes cancer cell proliferation and migration through epithelial–mesenchymal (EMT) transition. This gastrointestinal tumor progression is driven by upregulating epiregulin (EREG) and interleukin-11 (IL-11) [59] and chemotherapy resistance [60]. Furthermore, hypoxic in vitro and in vivo experimental models have revealed that the genetic suppression of estrogen receptor 1 (ESR1) in VASH-2-overexpressing, ESR1-positive cells leads to significant downregulation of E-cadherin expression. In addition, VASH-2 induces EMT in cancer cells by activating TGF-β1 and repressing the GATA3-ESR1 pathway under hypoxic conditions, thereby facilitating metastasis [61]. Notably, TGF-β1 is a potent inducer of IL-11 expression in stromal, epithelial, and cancer cells [62], and IL-11 has been implicated as a biomarker in various cancers and fibrotic diseases [63]. Several studies have shown that VASH-2 expression may serve as a prognostic biomarker. For example, VASH-2 is a biomarker for poor prognosis in pancreatic cancer [49]. Similarly, high VASH-2 expression is associated with poor prognosis and tumor growth in esophagus squamous cell carcinoma [48]. Additionally, the potential role of VASH-2 as a novel biomarker for diagnosis and prognosis has been confirmed in early stage lung squamous cell carcinoma [64]. These findings highlight a potential interplay between VASH-2, TGF-β1, and IL-11, suggesting shared upstream regulators and overlapping pathways in cancer progression and fibrosis. Further research is needed to elucidate the precise mechanisms underlying these relationships and their therapeutic implications.

### 3.2. Angiogenic Role of VASHs in Kidney Diseases

Chronic kidney disease (CKD) is characterized by a progressive loss of renal function, and dysregulation of angiogenesis is usually found to aggravate CKD development [65]; therefore, VASH-1 also plays a critical role in this process because it is a negative regulator of angiogenesis. For example, VASH-1 deficiency exacerbates cisplatin-induced acute kidney injury (AKI) due to the improper maintenance of peritubular capillary integrity following cisplatin-induced EC stress [66,67]. This suggests a protective role for VASH-1 in AKI by preserving vascular structure and function [66]. In contrast, elevated VASH-1 exacerbates disease progression, likely via TGF-β-driven fibrosis despite its protective role in acute injury [17,68]. This paradox may arise from hypoxia-induced pathway crosstalk in chronic disease, where prolonged anti-angiogenic signaling alters TGF-β responsiveness. These opposing outcomes highlight the context-dependent dual nature of VASH-1, where its anti-angiogenic properties can be either protective (e.g., in AKI) or detrimental (e.g., in DN), depending on the disease pathophysiology. Elevated plasma and urinary levels of VASH-1 and the VASH-1-SVBP complex were significantly correlated with worse renal consequences [69], further underscoring its dual role as both a protective agent and as a biomarker of progression in CKD [69].

On the other hand, VASH-2 has also been studied in mutant mice in response to ischemia-reperfusion (I/R) during AKI. VASH-2 knock-out mice showed more severe renal dysfunction and tubular damage after I/R injury, with elevated oxidative stress, apoptosis, neutrophil infiltration, and loss of peritubular capillaries, suggesting a protective reparative role via pro-angiogenic activity [70]. In addition, VASH-2 supports tubular repair in acute I/R injury, and its pro-angiogenic function worsens glomerular lesions in DN, highlighting context-dependent outcomes [70]. However, current evidence for the protective role of VASH-2 in I/R relies on knockout models; therefore, future studies need to explore exogenous VASH-2 administration to exclude developmental compensations. VASH-2 expression is localized to glomerular mesangial cells and is upregulated in the diabetic kidney in DN. These findings suggest that endogenous VASH-2 exacerbates DN, possibly by promoting angiogenesis, mesangial matrix expansion, and glomerular endothelial dysfunction. Thus, VASH-2, as a pro-angiogenic factor, contributes to glomerular lesions in DN, and its inhibition may be a potential therapeutic strategy for glomerular dysfunction [67]. In general, these divergent roles of VASH-1 and VASH-2 underscore the importance of context—including the disease type, duration, and microenvironment—to determine outcomes. Future studies are needed to clarify whether these paradoxes are kidney-specific or extend to other systems and to explore therapeutic strategies targeting VASHs.

### 3.3. Angiogenic Role of VASHs in Pathophysiology of the Reproductive System

Angiogenesis plays an important role in the development and function of the reproductive system and its organs [71,72]. There are multiple regulatory factors and mechanisms responsible for angiogenesis regulation [73]. Therefore, there is a need for a balance between pro-angiogenic factors that promote blood vessel growth and anti-angiogenic factors that inhibit it. Disruption of this balance leads to abnormal development of placental vasculature [74,75]. VASH-1 is specifically localized and expressed in ECs at the site of angiogenic initiation in certain pathophysiological processes of the human placenta [76], whereas VASH-2 plays a role in placental trophoblast differentiation and invasion [55]. VASH-2 overexpression promotes cell fusion during syncytiotrophoblast formation, and VASH-2 knockdown inhibits cell fusion [77]. Overall, VASH proteins enable the precise control of blood vessel growth required for optimal placental function by balancing the roles of the angiogenesis inhibitor (VASH-1) and angiogenesis promoter (VASH-2).

Angiogenesis also accompanies the establishment of pregnancy during corpus luteum (CL) development; therefore, large amounts of pro-angiogenic or anti-angiogenic factors play an important role in the CL [78,79]. For instance, prostaglandin F2 alpha-induced VASH-1 expression inhibited hyper-angiogenesis in early bovine CL and was drastically downregulated during mid-CL [80]. This was further confirmed when Shirasuna et al. [81] found the predominant location and expression of VASH-1 on the luteal ECs of bovine CL. HIF1-α is another key regulator in promoting ovarian angiogenesis in the CL in response to a hypoxic environment due to rapid luteal growth [82]. HIF1-α and VASH interplay perform pro-angiogenic and anti-angiogenic functions in CL formation and maturation, as well as in the ovarian follicle and in sustaining progesterone production [83]. Interestingly, VASH-1 has a pro-angiogenic role in diabetic erectile dysfunction, indicating it has an opposite role in the male reproductive system [84,85,86]. VASH-1 injection enhanced intracavernous angiogenesis, ultimately reversing erectile dysfunction [57,87]. This distinctive feature of VASH-1 lays the groundwork for future studies of erectile dysfunction mechanisms and treatments.

## 4. VASHs Serve as Tubulin Detyrosination Enzymes

The VASH-SVBP complex was recently found to play a divergent role in microtubule detyrosination [3]. The VASH-1 and SVBP complexes prioritize the detyrosination of the microtubule network (MTN) at the global level, whereas the VASH-2-SVBP complex plays a role in MTN detyrosination locally [27]. Although VASH-1 requires SVBP binding and VASH-2 acts in a self-governing manner with respect to detyrosination, SVBP is a bona fide activator of both of these enzymes [88]. The reversal of detyrosination is termed tyrosination, and both tyrosination and detyrosination are important biomarkers of dynamic and stable microtubules, respectively [89]. Dynamic instability is an essential property of microtubules that allows them to adapt to several critical functions, including cell division, the maintenance of cell shape, intracellular transport, and cell motility [90,91]. Therefore, the aberrant expression of VASHs can lead to MTN tyrosine and cytoskeletal abnormalities that can trigger the onset of a variety of diseases, such as neurological and cardiovascular diseases.

### 4.1. Detyrosinizing Role of VASHs in Neuronal Disorders

**Figure 4:**
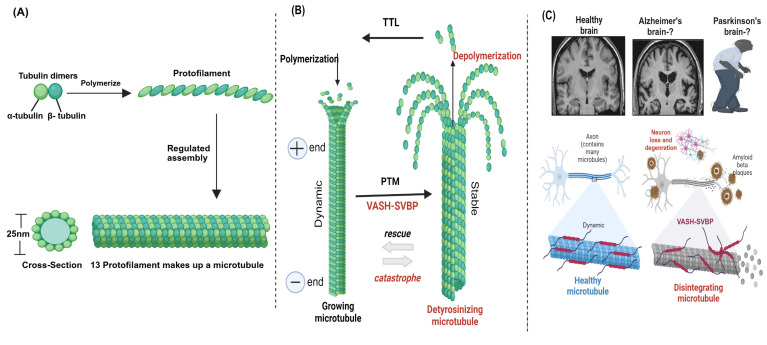
Microtubule structure, assembly, and post-translational modifications (PTMs) in the brain. (**A**) Microtubules are hollow cylindrical structures composed of α- and β-tubulin heterodimers [4]. (**B**) VASHs detyrosinate the microtubule through PTMs. (**C**) VASHs detyrosinate and disintegrate microtubules in the brain, leading to disease pathogenesis. VASH-mediated detyrosinated microtubules are involved in brain deformity [98], which may play a role in Alzheimer’s and Parkinson’s diseases. Created in biorender.com.

Proper tyrosination of microtubules is required for long-range transport, which has been demonstrated in neurons where an intact tyrosination cycle is required for neuronal organization and differentiation [10,92]. Similarly, a balance between dynamic and stable microtubules is required for neuronal survival and plasticity, but a shift in this balancing act can lead to brain degeneration [93,94]. Additionally, microtubule dynamics play a major role in the pre- and post-synaptic fragments of the synapse; therefore, intra-spinal microtubule dysregulation may lead to damaging results [95,96]. Neurodegeneration occurs in response to perturbations in the tyro-/detyrosination cycle, as it is strongly linked to microtubule dynamics [89,97]. SVBP deficiency in humans can lead to pathogenic neurodevelopment [98], and rare SVBP biallelic variants were found to induce defects in the brain associated with mental retardation [99]. Furthermore, VASH-1/2 inhibition accelerates the recovery of damaged nerves [100]. Taken together, these studies suggest that VASHs may play a role in brain-related diseases, particularly in Alzheimer’s disease, and may represent a novel therapeutic approach for treating brain-related diseases (Figure 4).

### 4.2. Detyrosinizing Role of VASHs in Cardiac Diseases

Heart diseases account for 17.9 million deaths annually and are the leading cause of death, posing significant challenges due to increased healthcare expenses [101,102]. Technological advances have helped to elucidate the role of microtubules in heart pathogenesis [103]. However, limited treatment approaches are aimed at microtubule-related mechanisms for cardiac diseases. Thus, we focus on the future perspective of treating heart pathologies with therapies that target microtubule-based mechanisms. Recent developments have spotlighted the role of the MTN in the mechanisms underlying heart malfunction [11]. The MTN of cardiac myocytes has certain architectural and biophysical characteristics that are necessary to meet the needs of the working heart [104]. The MTN is orientated towards the nucleus and aligns longitudinally along the myofibrillar matrix [105]; moreover, and it is assumed to serve as a dynamic transport mechanism surrounding mitochondria and along the plasma membrane in cardiac myocytes [106]. Pathological cardiac remodeling is characterized by changes in MTN density, stability, and PTMs. Therefore, altered microtubules may directly impair cardiomyocyte contractile performance in various cardiac diseases [107]. Increased cellular MTN density explains a significant proportion of the cardiomyocyte contractile failure associated with pressure-overload-induced cardiac hypertrophy [108]. Moreover, mRNAs and ribosomes are transported to aid in local translation and to assemble contractile units. Rather than the translation rate, which is known to be a critical factor of cardiac hypertrophy, proper localized translation was suggested to be a factor of cardiac hypertrophy. Evidence suggests that microtubule-based transport augments the amplified transcription and translation for the effective growth of cardiomyocytes during cardiac stress [109]. Similarly, VASH-1 acts as a hypoxia-responsive IRES trans-acting factor in cardiomyocytes, with its ischemic heart role still undetermined [110].

**Figure 5:**
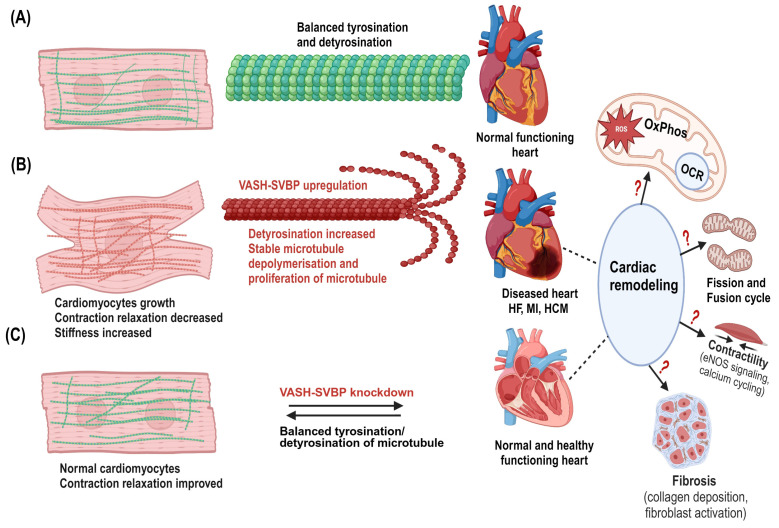
Potential mechanisms of VASHs in the cardiac system. (**A**) Normal cardiomyocytes with dynamic microtubules (green in color) and balanced tyrosination/detyrosination cycle in which the heart performs normal functions. (**B**) Cardiomyocyte growth occurs particularly in the diseased hypertrophic heart. VASH-SVBP may highly detyrosinate, stabilize, and proliferate microtubules. Denser microtubules (red) are seen in cardiomyocytes. Microtubules are in proximity to mitochondria in cardiomyocytes; therefore, upregulation of VASHs could lead to mitochondrial dysfunctions in response to cardiac remodeling. (**C**) VASH-SVBP suppression may improve contraction and relaxation, as well as decrease cardiac microtubule dysfunction, hence, balance between tyrosinated/detyrosinated states, representing the normal/healthy cardiomyocytes and heart. This remodeling could also alter both mitochondrial function and dysfunction. Thus, it is critical to study these alterations and mechanisms, including bioenergetics, fission and fusion, and fibrosis in the future. Created in biorender.com.

Cardiac microtubules provide viscoelastic resistance to myofilament shortening and re-lengthening by physically coupling to myofilaments. This interaction is regulated by detyrosination, which is one of the major microtubule PTMs [111,112]. Cardiomyocytes obtained from heart failure patients have a denser MTN, which is also much more detyrosinated than that observed in healthy hearts [113]. Due to these MTN modifications, the contraction–relaxation cycle is slower when compared to that of healthy cardiomyocytes [114,115]. VASH/SVBP plays a significant role in the detyrosination of cardiac microtubules in a failing heart. Briefly, VASH-1 depletion causes a decrease in stiffness and enhancement of cardiac microtubule contractile performance in cardiomyocytes from heart failure patients with preserved or reduced ejection fraction [116]. During this process, the phosphorylation of microtubule-associated protein 4 by microtubule-affinity-regulating kinase 4 (MARK4) gives VASHs more access to detyrosinating α-tubulin, and the loss of ejection fraction is markedly reduced in the absence of MARK4 in an acute myocardial infarction model [117]. Therefore, VASH-1 upregulation stabilizes microtubules by detyrosination, signifying its potential therapeutic role in cardiac hypertrophy (Figure 5).

## 5. The Pathophysiological Role of VASHs in Fibrosis

**Figure 6:**
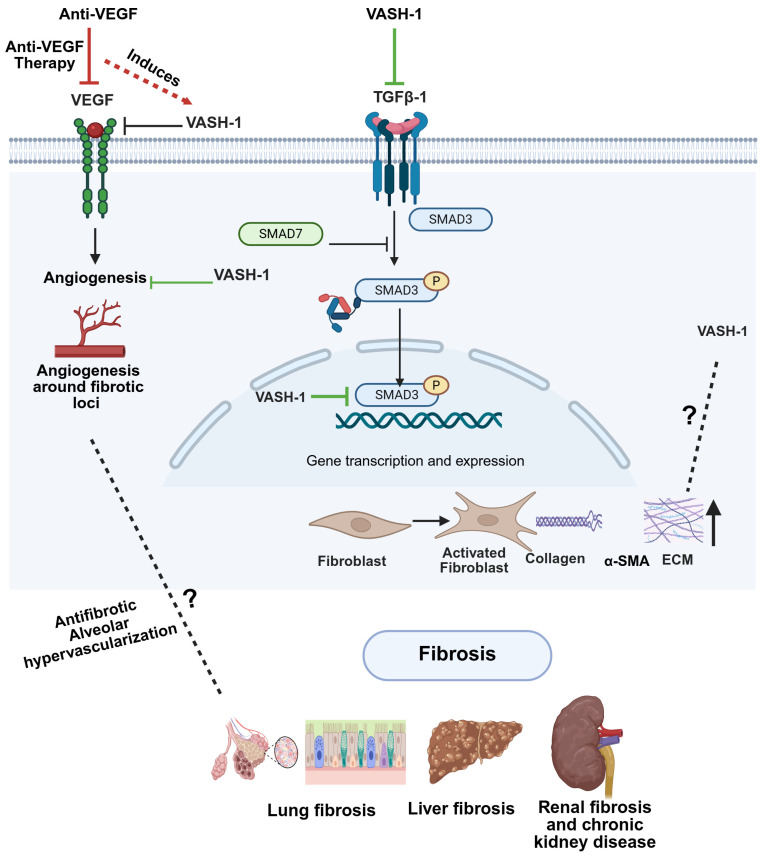
Potential mechanistic interplay between VASH-1, VEGF, and TGF-β signaling in fibrosis. Anti-VEGF therapy upregulates VASH-1, which subsequently inhibits both VEGF signaling (negative feedback) and TGF-β1/SMAD3 activation. (**Right**) This dual inhibition by VASH-1 reduces angiogenesis around fibrotic loci and could block fibroblast activation, collagen deposition, and ECM remodeling. While VASH-1 inhibits TGF-β signaling, the potential regulation of VASH-1 by TGF-β remains undefined. Created in biorender.com.

Beyond their role in angiogenesis and detyrosination, VASHs are also involved in regulating fibrosis [118] (Figure 6). Fibrosis is a disease associated with excessive buildup of ECM, which causes stiffness and progressive scarring that can lead to organ dysfunction and, ultimately, death [119]. Fibrosis development is regulated critically by the TGF-β1/SMAD3 signaling pathway, which can be modulated by VASHs. For example, VASH-1 has been shown to modulate TGF-β1 signaling in the kidney, providing a protective role against ECM formation, renal inflammation, and fibrosis [120]. Diabetic kidney disease (DKD) is characterized by a slow progression of persistent proteinuria that eventually leads to renal failure, and two major pathogenic changes in DKD are fibrosis and oxidative stress [121,122]. While VASH-1 expression is suppressed during DKD progression, its residual activity may partially restrain TGF-β1/Smad3-driven fibrosis, as VASH-1 depletion exacerbates renal damage via unchecked TGF-β1 signaling and amplifies oxidative stress through dysregulated SIRT1/HIF1-α pathways [118]. This paradoxical role, where VASH-1 is downregulated yet mechanistically implicated in mitigating DKD progression, highlights its potential as a therapeutic target. Restoring VASH-1 activity could, thus, simultaneously target fibrosis and oxidative stress, two hallmarks of DKD pathogenesis.

VASHs have the potential for various therapeutic uses, from regulating angiogenesis and fibrosis to playing a major role in detyrosination. Based on these insights, the way forward requires intensive efforts to explore VASH biology and pathophysiology while translating these discoveries into clinical applications. The structural mechanisms underlying VASH isoform specificity—particularly how VASH-1 and VASH-2 differentially engage angiogenesis and microtubule pathways—demand an explanation through high-resolution cryo-EM studies. Simultaneously, we must develop targeted therapeutic strategies for the tissue-specific effects, such as blood–brain-barrier-impermeable formulations to protect neuronal microtubules while permitting peripheral VASH modulation. Moreover, many aspects of VASHs, especially their interplay in regulating angiogenesis, fibrosis, and detyrosination, may be further explored in future studies.

In addition to regulating TGF-β1/SMAD3 signaling, VASHs may influence fibrosis via angiogenesis. Most chronic liver diseases, including hepatocellular carcinoma, are characterized by fibrosis that eventually progresses to cirrhosis [123]. Chronic liver disease development is linked to pathological angiogenesis, and angiogenesis suppression has been shown to attenuate liver fibrosis in bile duct ligation and carbon tetrachloride mouse models [124,125]. It has been proposed that anti-angiogenic therapeutics may prevent liver fibrosis [126,127]. Indeed, VASH-1 was found to inhibit fibrosis and cirrhosis in rat liver [128]. Because VASH-1 is an anti-angiogenic and antifibrotic protein, it is a promising therapeutic approach that can treat fibrosis and protect the liver with high efficacy. VEGF-A and FGF are involved in pulmonary fibrosis [129], which develops in response to vascular remodeling in the lungs [130]. VEGF overexpression increases permeability in the pulmonary vasculature, leading to edema [131]. Anti-VEGF therapy reduces lung injury and fibrosis [132,133]. Furthermore, VASH-1 attenuated pulmonary fibrosis through its anti-angiogenic activity and a significant decrease in cytokine secretion, lymphocyte infiltration, and fibroblast proliferation [134].

## 6. Conclusions and Future Remarks

**Figure 7:**
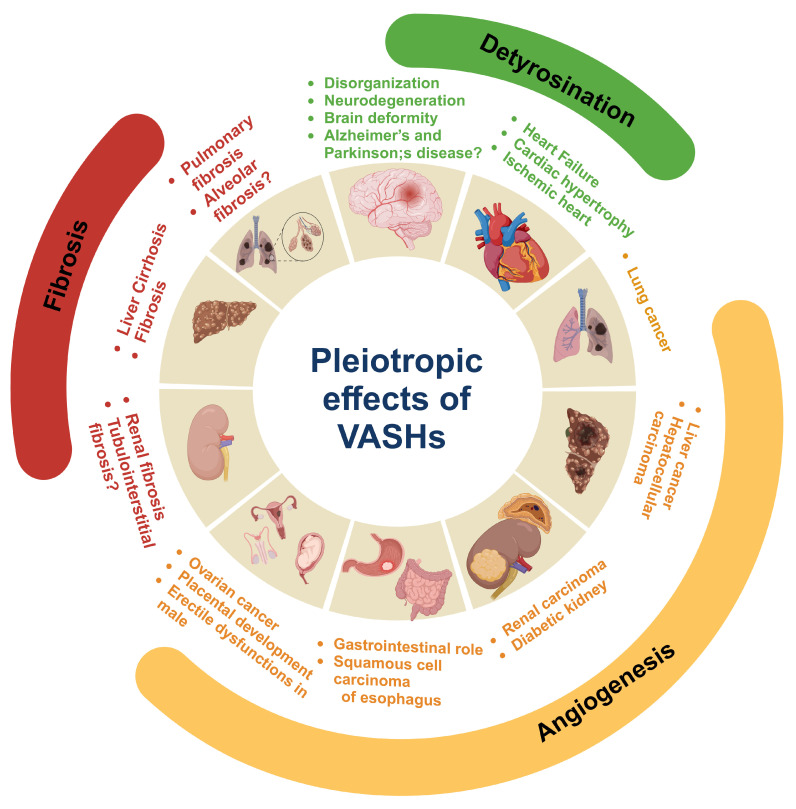
Pleotropic role of vasohibins (VASHs) in health and disease. VASHs play key roles in neurodegeneration and disorganization in neurons of the brain by inducing microtubule detyrosination. VASHs detyrosinate cardiac microtubules and impede contraction and relaxation. Therefore, the suppression of VASHs aids in the kinetics of contraction, which ultimately improves cardiac function and could have therapeutic use in heart failure, ischemic heart disease, and cardiac hypertrophy. In angiogenesis, VASH-1 plays an anti-angiogenic role, whereas VASH-2 is pro-angiogenic. The regulation of VASHs during angiogenesis plays a critical role in tumor growth and maintaining the functions of the gastrointestinal tract, as well as male and female reproductive organs. Moreover, the regulatory role of VASHs has been documented in liver, kidney, and pulmonary fibrosis. Created in biorender.com.

As summarized in Figure 7, VASHs are pleiotropic molecules that are regulated by VEGF, which in turn inhibit the VEGF pathway and, thus, the process of angiogenesis. Therefore, the abnormal expression of VASHs could trigger various diseases related to angiogenesis, such as cancer, kidney disease, and reproductive diseases. Beyond their well-characterized role in angiogenesis, recent studies have revealed that VASHs have cysteine protease activity, can regulate the detyrosination of α-tubulin, and modulate the dynamic changes in microtubules. In addition, VASHs can inhibit fibrotic lesions in multiple organs through mediating the TGF-β1/SMAD3 pathway and angiogenesis. However, many of the biological functions and molecular mechanisms of VASHs remain unknown, including the following: (1) the molecular mechanism by which VASHs regulate VEGFR2 expression and phosphorylation; (2) the molecular interactions between VASHs and the TGF-β1/SMAD3 pathway; (3) whether VASHs regulate angiogenesis and fibrosis through modulating microtubules; (4) the divergent role and tissue specificity of VASH-1 and VASH-2 in microtubule dynamic regulation; (5) the level at which VASHs detyrosinate the microtubule subset population, which can enhance its therapeutic index; and (6) whether VASHs are involved in the regulation of cardiac cells in the heart, given their regulation of MTNs, which are tightly connected to the mitochondrion morphology and function of the heart, an oxygen-demanding organ where cardiac cells have mitochondria that occupy 30% of their volume [135,136].

The abnormal expression of VASHs is closely related to the occurrence and development of various diseases, according to many studies, but their potential as a therapeutic target remains to be evaluated. Anti-VEGF therapy has been a highly anticipated treatment strategy for various diseases, including cancer, but it has faced clinical challenges due to various undeniable side effects. Because VASHs are a negative feedback molecule of VEGF with relatively higher specificity, they have the potential to become effective targets for anti-angiogenic therapy. However, VASHs have a recently discovered role in microtubule regulation; therefore, targeting VASHs may lead to unknown side effects due to microtubule disruption, which in turn affects the cytoskeleton and cellular architecture. Therefore, a deeper study of the functions and mechanisms of VASHs can provide a solid foundation for therapeutically exploiting VASHs for the treatment of angiogenesis-related diseases. The integrated perspective serves as both compass and caution, guiding therapeutic development while reminding us of the critical balance between VASH’s pleiotropic benefits and its potential to disrupt fundamental cellular architecture. Through interdisciplinary collaboration, we can advance VASH modulation from mechanistic curiosity to meaningful clinical impact.

## Data Availability

The original contributions presented in this study are included in the article/Supplementary Materials. Further inquiries can be directed to the corresponding author.

## Supplementary Materials

The following supporting information can be downloaded at: https://www.mdpi.com/article/10.3390/cells14110767/s1, Table S1. The vasohibin (VASH) protein sequences (obtained from NCBI, accession numbers provided in the Supplementary File.

## Abbreviations

VASHs	Vasohibins
VEGF	Vascular endothelial growth factor
ECs	Endothelial cells
TCP	Tubulin carboxypeptidase
PTM	Post-translational modification
TTL	Tubulin tyrosine ligases
SVBP	Small vasohibin-binding protein
AA	Amino acids
FGF-2	Fibroblast growth factor-2
TGF	Transforming growth factor
ECM	Extracellular matrix
PKC-δ	Protein kinase C-delta
TNF-α	Tumor necrotic factor-alpha
IL-1β	Interleukin-1beta
IFN-γ	Interferon-gamma
HIF1-α	Hypoxia inducible factor 1 alpha
CKD	Chronic kidney disease
AKI	Acute kidney injury
DN	Diabetic nephropathy
I/R	Ischemia-reperfusion
CL	Corpus luteum
MTN	Microtubule network
MARK4	Microtubule-affinity-regulating kinase 4
MI	Myocardial infarction
DKD	Diabetic kidney disease
SMAD3	Mothers against decapentaplegic homolog 3
